# Mental health of people with limited access to health services: a retrospective study of patients attending a humanitarian clinic network in Germany in 2021

**DOI:** 10.1186/s12888-023-04727-7

**Published:** 2023-04-19

**Authors:** Kashung Annie Mugambwa, Wandini Lutchmun, Janina Gach, Carolin Bader, Guenter Froeschl

**Affiliations:** 1 Division of Infectious Diseases and Tropical Medicine, University Hospital, LMU Munich, Munich, Germany; 2 Center for International Health, Ludwig-Maximilians-Universität, Munich, Germany; 3 Ärzte der Welt Deutschland e.V, Munich, Germany

**Keywords:** Germany, Mental health, Health access, Humanitarian aid, Migrants, Homelessness

## Abstract

**Background:**

Germany has a statutory health insurance system. However, a substantial part of the population still suffers from limited access to regular health services. While humanitarian organizations are partially filling this gap, people with limited access show a high prevalence of mental disorders. This study investigates the prevalence, and social determinants of mental disorders in patients attending the clinics of a humanitarian health network in three major cities in Germany, as well as perceived barriers to healthcare access in this population.

**Methods:**

We performed a descriptive, retrospective study of individuals attending the outpatient clinics of the humanitarian organization Ärzte der Welt, in Berlin, Hamburg and Munich, in 2021. Medico-administrative data was collected using a digital questionnaire at first presentation to the clinics. We report the prevalence of both perceived altered mental health and diagnosed mental disorders, as well as the perceived barriers to healthcare access in this population. We performed a logistic regression analysis to identify the socio-demographic factors associated with mental disorders.

**Results:**

Our study population consisted of 1,071 first presenters to the clinics in 2021. The median age at presentation was 32 years and 57.2% of the population were male. 81.8% experienced a form of homelessness, 40% originated from non-EU countries and only 12.4% had regular statutory health insurance. 101 (9.4%) patients had a diagnosed mental disorder. In addition, 128 (11.9%) patients reported feeling depressed, 99 (9.2%) reported a lack of interest in daily activities, and 134 (12.5%) lacked emotional support in situations of need on most days. The most reported barrier to accessing health services was high health expenses, reported by 61.3% of patients.In the bivariate logistic regression analysis age, insurance status and region of origin were significantly associated with mental disorders. In the multivariable analysis, only age groups 20–39 and 40–59 years remained significant.

**Conclusions:**

People with limited access to regular health services have a high need for mental health services. As a chronic condition, this is even more difficult to manage outside of regular services, where humanitarian clinics are only filling the gap in serving basic health needs.

## Introduction

Mental disorders account for about 14% of the global burden of diseases [1, 2] According to the World Health Organization (WHO), one in four people worldwide suffers from a mental disorder [3] In Germany, more than a quarter of the adult population is affected by mental illness each year, the most common being anxiety disorders, mood disorders, and disorders caused by alcohol and drug consumption [4].

The probability of developing amental disorder in one’s lifetime is shaped by multiple social, economic, and environmental factors, acting at various phases of life [5]. Adverse childhood experiences, inadequate and unequal education, food insecurity, unstable housing, unemployment, restricted access to healthcare, poverty and discrimination are few examples of socio-economic factors that can influence mental health outcomes [6]. Exposure to a severe traumatic event, for example, may result in trauma-related disorders, such as post-traumatic stress disorder (PTSD), chronic pain syndrome, or other psychosomatic syndromes [7]. Additionally, a 2014 WHO report on social determinants of health suggests that a lack of participation in social activities can contribute to poor health, and potentially exacerbate physical disease and mental disorders [8, 9].

Refugees and migrants constitute a particular risk group due to exposures linked to migration trajectories and living conditions. While navigating resettlement and undergoing regularization processes in their host countries, many refugees and migrants lack access to health promotion, disease prevention, and mental health services, in addition to treatment, care, and financial protection [10].

Furthermore, other factors such as homelessness and incarceration are frequent occurrences for people with mental disorders, exacerbating their marginalization and exclusion. Rates of mental disorders among persons experiencing homelessness (PEH) can be greater than 50%, and studies have reported more than one third of prison populations with mental disorders [11, 12].

According to the WHO, many mental disorders can be effectively treated at relatively low cost [13], yet the gap between the need for treatment and its provision is wide all over the world [14]. Estimates show that between 35% and 50% of people with severe mental disorders in high-income countries receive no treatment; the corresponding range for low- and middle-income countries has been between 76% and 85% [15]. Adding to the complexity of the problem, people with limited access to health services are a difficult to reach population. This is partly because it is a very heterogeneous group, comprising undocumented migrants, persons living in substandard living conditions and other persons experiencing social exclusion and marginalisation [15, 16]. This is equally true for high-income-countries, such as Germany [17]. This study investigates the prevalence of mental disorders and examines socio-demographic factors contributing to their development in patients seeking medical care at three humanitarian outpatient clinics in Berlin, Hamburg, and Munich, the three largest cities in Germany.

## Methods

### Objectives

This study describes the prevalence of mental disorders in persons with limited access to regular healthcare services who sought care at the clinics of a humanitarian organisation in 2021 in Germany. Furthermore, we describe the socio-demographic characteristics and perceived barriers to accessing healthcare services in this population.

### Setting

Ärzte der Welt, the German branch of the organisation Médecins du Monde, was founded in 2000. Domestic healthcare programs are carried out in cooperation with local partner organizations in Hamburg, Berlin, and Munich through the so-called “open.med clinics” (drop-in centres) and an additional mobile clinic that circulates in social hot spots in Munich. The humanitarian organisation provides charitable medical care to disadvantaged people with limited access to the healthcare system in Germany. The services in Munich and Berlin comprise scheduled consultation hours by a trained psychiatrist and psychotherapist. The focus of the projects is the (re-) integration of the patients into the regular healthcare system, and to politically enforce the right to healthcare. Therefore, the organization provides both medical care and social counselling to individuals presenting to the clinics and mobile projects. As part of the advocacy work of Ärzte der Welt, an annual openly published report is made available to political decision-makers, stakeholders in health, and other welfare associations.

### Study population

The study population consisted of 1,071 patients who presented to the humanitarian clinics and mobile unit in Berlin, Hamburg, and Munich for the first time in 2021. Patients either self-presented or were referred by elements of the regular healthcare services, other civil society or welfare projects and local support networks, including friends and family.

### Study design

We performed a retrospective analysis of the full health datasets of all individuals who presented to the three humanitarian clinics and mobile unit of Ärzte der Welt and its partners in Berlin, Hamburg, and Munich in 2021. The data was entered by a healthcare worker into a web-based digital database. The data was extracted, irreversibly anonymized, and then incorporated into a Microsoft Excel spreadsheet.

### Measures and variables

This paper describes two measures of altered mental health. “Perceived altered mental health” is a subjective measure and indicates how patients responded to the following questions during counselling sessions at the clinics : “Over the last 2 weeks, how often have you felt down, depressed, or hopeless?”, “How often do you have little interest or pleasure in doing things?” and “How often do you receive emotional support from someone or having a person who can offer help and trust when needed?” Possible answers to these questions were: “not at all”, “on several days”, “more than half the days”, and “nearly every day”. Feelings of depression and low mood were denoted as the variable “perceived depression”; a lack of interest in performing daily activities as “lack of interest”: and a perceived availability of moral support as “moral support”. For the purpose of the logistic regression analysis, “perceived depression” and “lack of interest” were dichotomised into “more often”, which included the answers “nearly every day” and “more than half the days”, and “less often” which included “not at all” and “on several days”. The variable “moral support” was dichotomized into “lack of moral support” (can never, or only sometimes, rely on someone for moral support) and “moral support available” (can often or very often rely on someone for moral support).

The other measure of altered mental health were medical diagnoses of mental disorders inputted by a health professional at consultation, using the International Classification of Diseases, Tenth Revision (ICD-10). The variable **“**mental disorder” was generated by matching all ICD-10 codes within the group “F” (behavioural/mood and mental disorders). The variable “ mental disorder” was dichotomized into 1 = individuals with a mental disorder and 0 = individuals with no mental disorders stated.

The screening tools for mental health were selected based on their validity and reliability in a clinical setting. All healthcare professionals performed an assessment interview or administered a psychological questionnaire to explore the occurrence of common depressive symptoms in the last 14 days.

Socio-demographic characteristics as independent variables were categorised as follows: age was categorised into 4 groups: < 20 years; 20–39 years; 40–59 years; and > = 60 years. Housing situation was categorised as roofless (without shelter of any kind or sleeping rough), houseless (having a place to sleep but temporary in institutions or shelter), insecure housing (without secure tenancy, living with friends/family, or at risk of eviction), inadequate housing (living in caravans on illegal campsites), and secure housing (having a house or apartment secured by a tenancy agreement). In the logistic regression analysis, housing situation was dichotomised into “precarious housing” (which encompassed all forms of homelessness and included roofless, houseless, insecure, and inadequate housing) and “non-precarious housing” which included persons with secure housing.

Finally, perceived barriers, which encompassed migration background, social, economic, language and health dimensions, were selected based on findings from previous studies in this domain. During the social counselling sessions at first visit to the clinics, patients were asked by a social worker about the perceived barriers that hindered their access to healthcare services. We describe these perceived barriers in patients with diagnosed mental disorders.

### Statistical analysis

Descriptive categorical data are presented in proportions, the continuous variable age in median and range. Correlations between categorical variables were analysed by applying the chi²-test. The threshold of statistical significance was set at α = 0.05.

A logistic regression analysis was performed to identify socio-demographic factors associated with mental disorders. The main outcome in both bivariate and multivariable analyses was having a diagnosed mental disorder. We first conducted bivariate analyses between all variables and the main outcome and retained factors with a *p*-value of 0.2 or lower. We then fitted three regression models whose results are presented as adjusted odds ratio (aOR) with 95% CI. The first model included sociodemographic and mental disorder-related variables.

All statistical analyses were performed using Stata SE 17 (StataCorp, College Station, USA).

## Results

### Socio-demographic characteristics

In 2021, 1,071 patients presented to the clinics for the first time, of whom 57.2% (611/1,069) were male and 42.8% (458/1,069) were female. The median age at presentation was 32 years (IQR: 6.4–46.0 years; range zero to 84). Overall, 40.0% (424/1,059) of patients originated from non-EU countries, 39.9% (423/1,059) from EU/EEA countries and 20.0% (212/1,059) were German nationals. Among those with known housing status and health coverage, most patients lived in precarious housing (81.8%; 775/947), of whom the majority (30.7%; 291/947) were houseless. Only 12.4% (115/930) had regular statutory or private insurance.

Of all patients that gave information on their private economic situation, 98.0% (869/887) stated to dispose of an income below the current income poverty line for Germany, which is € 1,136.- per month.

### Mental disorders

Multiple diagnoses could be made in single patients. Of 1,691 total diagnoses that were made in 1,071 patients at first visits to the clinics, 101 (5.9%) fell into the category of a behavioral/mood disorder. Since we intend to highlight the range of all mental disorders, the denominator in this paragraph is the number of mental disorder diagnoses made. Within this group, the most predominant mental disorders were reaction to severe stress and adjustment disorders/post-traumatic stress disorder (PTSD) (16.8%; 17/101), depressive episode (14.9%; 15/101), alcohol-related disorders (8.9%; 9/101), other anxiety disorders (7.9%; 8/101), somatoform disorders (7.9%; 8/101), and sleep disorders not due to a substance or known physiological condition (6.9%; 7/101). A high prevalence of mental disorders (52.5%; 52/99) was found in the age group 20–39 years, followed by the age group 40–59 years (32.3%; 32/99). Table 1 describes the socio-demographic characteristics of patients with and without a mental disorder who attended the clinics in 2021.

**Table 1:** Socio-demographic characteristics of patients with mental disorders and patients without mental disorders, reported as number and percentage of diagnoses

Socio-demographic characteristics	Number of mental disorders s N (%)	Number of non- mental disorder diagnoses N (%)	P value*	Total number of participants N (%)
**Gender**			< 0.005	
Male	61.0% (61/100)	56.4% (894/1,585)		57.2% (611/1,069)
Female	39.0% (39/100)	43.6% (691/1,585)		42.8% (458/1,069)
**Age category**			< 0.005	
< 20 years	8.1% (8/99)	32.9% (509/1549)		32.7% (341/1,042)
20–39 years	52.5% (52/99)	28.2% (436/1549)		32.0% (333/1,042)
40–59 years	32.3% (32/99)	27.6% (427/1,549)		26.4% (275/1,042)
>= 60 years	7.1% (7/99)	11.4% (177/1,549)		8.9% (93/1,042)
**Nationality**			0.015	
German nationals	13.3% (13/98)	22.2% (351/1,579)		20.0% (212/1,059)
EU/EAA	34.7% (34/98)	39.5% (624/1,579)		39.9% (423/1,059)
Non-EU	52.0% (51/98)	38.3% (604/1,579)		40.0% (424/1,059)
**Insurance**			< 0.005	
Fully insured	7.4% (7/94)	12.7% (179/1,408)		12.4% (115/930)
Partially insured	10.6% (10/94)	11.4% (160/1,408)		10.8% (100/930)
Not Insured	83.0% (77/94)	75.9% (1,069/1,408)		76.9% (715/930)
**Housing Situation**			0.156	
Secure	18.2% (18/99)	16.4% (233/1,420)		18.2% (172/947)
Houseless	19.2% (19/99)	31.7% (450/1,420)		30.7% (291/947)
Inadequate	2.0% (2/99)	1.6% (23/1,420)		1.7% (16/947)
Insecure	35.3% (35/99)	29.2% (414/1,420)		29.4% (279/947)
Roofless	25.3% (25/99)	21.1% (300/1,420)		20.0% (189/947)
**Immigration status**			0.031	
EU citizens over 3 months stay with no entitlements	4.4% (4/90)	6.6% (91/1,375)		6.6% (60/913)
EU citizens over 3 months stay with entitlements	16.7% (15/90)	15.1% (207/1,375)		15.3% (140/913)
EU citizens under 3 months stay with entitlements	15.6% ( 14/90)	16.7% (230/1,375)		16.6% (152/913)
German nationals	14.4% (13/90)	25.3% (348/1,375)		22.9% (209/913)
Non- EU citizens with an asylum claim	5.6% (5/90)	2.3% (31/1,375)		3.1% (28/913)
Non-EU with no asylum claim	7.8% (7/90)	3.3% (45/1,375)		3.5% (32/913)
Non-EU undocumented migrants	21.1% (19/90)	15.2% (209/1,375)		16.4% (150/913)
Non-EU refugees	3.3% (3/90)	1.0% (15/1,375)		1.3% (12/913)
Non-EU legal migrants	11.1% (10/90)	14.5% (199/1,375)		14.3% (130/913)
**Clinics location**			0.054	
Berlin	39.6% (40/101)	39% (620/1,590)		41% (438/1,071)
Hamburg	4% (4/101)	11.9% (190/1,590)		12.8% (137/1071)
Munich	33.6% (34/101)	32.7% (520/1,590)		29.9% (321/1,071)
Munich mobile clinics	22.8% (23/101)	16.4% (260/1,590)		16.3% (175/1071)

*P value refers to the difference between mental disorder and no mental disorder determined using chi² test. Denominators are based on the number of diagnoses made and are varying due to missing data

### Factors associated with mental disorders

We performed a logistic regression analysis to identify the relationship between mental disorders and socio-demographic characteristics (Table 2). In the baseline model of the bivariate analysis, belonging to the age group 20–39 years (aOR: 7.53; 95% CI 3.53–16.05), 40–59 years (aOR: 4.70; 95% CI 2.13–10.34), originating from a non-EU country (aOR: 2.27; 95% CI 1.22–4.25), and being partially insured (aOR: 3.33; 95% CI 1.22–9.04), were significantly associated with higher odds of a mental disorder. Having full health coverage was significantly protective for the main outcome (aOR: 0.29; 95% CI 0.08–0.93).

In the multivariable analysis, belonging to the age groups 20–39 and 40–59 years was the only factor that remained significantly associated with increased odds of mental disorder, (OR: 3.13; 95% CI 1.21–8.11).

**Table 2:** Summary results of the bivariate and multivariable logistic regression analysis analysing socio-demographic factors associated with a higher likelihood of a mental disorder

Socio-demographic characteristics	Bivariate analysis		Multivariable analysis	
	aOR (95% CI)	P-value	aOR (95% CI)	P-value
**Gender** (reference: female)	1.00		1.00	
Male	1.24 (95% CI 0.83–1.82)	0.285	1.43 (95% CI 0.89–2.30)	0.131
Age group (reference: < 20)	1.00		1.00	
20–39 years	7.58 (95% CI 3.56–16.14	**< 0.005**	8.04 (95% CI 3.06–21.14)	**< 0.005**
40–59 years	4.76 (95% CI 2.17–10.45	**< 0.005**	6.56 (95% CI 2.52–17.12)	**< 0.005**
>= 60 years	2.51 (95% CI 0.89–7.03)	0.079	3.59 (95% CI 1.15–11.25)	0.028
**Nationality**				
German nationals (reference)	1.00		1.00	
EU/EEA	1.47 (95% CI 0.76–2.82)	0.246	0.69 (95% CI 0.32–1.47)	0.511
Non-EU	2.27 (95% CI 1.22–4.25)	**0.010**	1.14 (95% CI 0.32–3.95)	0.835
**Housing status**				
Precarious housing (reference)	1.00		1.00	
Non- precarious housing	1.13(95% CI 0.66–1.92)	0.646	1.26 (95% CI 0.70–2.26)	0423
**Insurance**				
Fully Insured (reference)	1.00		1.00	
Limited Insured	1.81 (95% CI 1.22–9.04)	**0.018**	1.32 (95% CI 0.55–3.17)	0.531

Since in the multivariable analysis, the age group 20–39 years of age remained significantly associated with a mental disorder and constituted a large share of patients, we decided to conduct a further subgroup analysis on this age group in order to control for possible lifetime period effects.

In this age group, comprising 333 individuals, 58.9% (196/333) were male, 57.9% (191/330) originated from a non-EU country, 78% (234/300) had no insurance coverage and 39% (112/287) were undocumented migrants.

The most prevalent mental disorders were depressive episode, reaction to severe stress and adjustment disorders, and anxiety disorders, 19.2% (10/52), 19.2% (10/52) and 13.5% (7/52) respectively.

### Subjective indicators of mental health needs

Table 3 describes socio-demographic characteristics of the population reporting a perceived altered mental health. Most notably, in the age group 20–39 years, 54.8% (69/126) individuals reported experiencing feelings of depression, 55.1% (54/98) reported a lack of interest in daily activities, and 44.3% (58/131) reported not receiving psychological support from a trusted person, friend, or family when in need, on most days of the week. Additionally, when stratified by region of origin, persons originating from non-EU countries also exhibited a high proportion of depressive feelings, a lack of interest in daily activities and social isolation. However, the association was not statistically significant (P- value: 0.322; 0.539 and 0.828 respectively).

Furthermore, a perceived altered mental health was reported in high proportion among persons with limited or no health coverage (87.8%), and 27.3% of non-EU undocumented migrants reported feeling depressed on many occasions.

**Table 3:** Socio-demographic characteristics of patients with and without perceived altered mental health, reported as number and percentage of patients

Socio-demographic characteristics	Self-reported feeling depressed N (%)		P-value	Lack of interest in daily activities N (%)		P-value	Moral support received from someone when needed N (%)		P-value
	More often	Less often		More often	Less often		No	Yes	
**Gender**			0.152			0.505			0.298
Male	50.8% (64/126)	51.9% (123/237)		48% (47/98)	45.8% (99/216)		59.7% (80/134)	46.8% (169/361)	
Female	49.2% (62/126)	48.1% (114/237)		52% (51/98)	54.2% (117/216)		40.3% (54/134)	53.2% (192/361)	
**Age group**			**< 0.005**			**< 0.005**			0.802
< 20 years	7.1% (9/126)	22.5% (52/231)		7.2% (7/98)	24.1% (51/212)		24.4% (32/131)	28.1% (100/356)	
20–39 years	54.8% (69/126)	45.4% (105/231)		55.1% (54/98 )	43.9% (93/212)		44.3% (58/131)	39.9% (142/356)	
40–59 years	29.4% (37/126)	26% (60/231)		27.5% (27/98)	26.4% (56/212)		23.7% (31/131)	23.6% (84/356)	
>= 60 years	8.7% (11/126)	6.1% (14/231)		10.2% (10/98)	5.6% (12/212)		7.6% (10/131)	8.4% (30/356)	
**Nationality**			0.322			0.539			0.828
German nationals	13.38% (17/127)	16.5% (39/237)		13.3% (13/98)	17,5% (38/217)		19.4% (26/134)	21.9% (79/361)	
EU/EAA	24.41% (31/127)	29.5% (70/237)		25.5% (25/98)	27.2% (59/217)		26.9% (36/134)	25.5% (92/361)	
Non-EU	62.2% (79/127)	54% (128/237)		61.2% (60/98)	55.3% (120/217)		53.7% (72/134)	52.6% (190/361)	
**Housing situation**			0.937			0.869			0.013
Secure	21.3% (27/127)	23.8% (54/234)		23.5% (23/98)	23.4% (50/214)		12.1% (16/132)	25.8% (93/360)	
Houseless	19.7% (25/127)	22.4% (53/234)		19.4% (19/98)	22.4% (48/214)		30.3% (40/132)	25.3% (91/360)	
Inadequate houses	2.4% (3/127)	2.6% (6/234)		1.1% (1/98)	2.8% (6/214)		3.8% (5/132)	1.7% (6/360)	
Insecure houses	47.2% (/127)	44.4% (104/234)		48% (47/98)	45.3% (97/214)		44.7% (59/132)	41.9% (151/360)	
Roofless	9.4% (12/127)	7.3% (17/234)		8% (8/98)	6.1% (13/214)		9.1% (12/132)	5.3% (19/360)	
**Insurance status**			0.635			0.663			0.179
Fully insured	12.2% (28/230)	10.5% (13/124)		14.4% (30/209)	12.5.4% (12/96)		18.5% (24/130)	13.5% (47/347)	
Limited insured	87.8% (202/230)	89.5% (111/124)		85.6% (179/209)	87.5% (84/96)		81.5% (106/130)	86.5% (300/347)	
**Immigration status**			0.013			0.099			0.619
EU citizens over 3 months stay with no entitlements	4.9% (6/121)	3.6% (8/222)		2.1% (2/96)	4% (8/200)		4.6% (16/343)	2.4% (3/125)	
EU citizens over 3 months stay with entitlements	14.9% (18/121)	12.2% (27/222)		16.7% (16/96)	12% (24/200)		12.8% (44/343)	12.8% (16/125)	
EU under 3 months stay with entitlements	6.6% (8/121)	17.1% (38/222)		7.3% (7/96)	15.5% (31/200)		10.8% (37/343)	13.6% (17/125)	
German nationals	14% (17/121)	17.6% (39/222)		13.5% (13/96)	19% (38/200)		22.7% (78/343)	20.8% (26/125)	
Non- EU citizens with an asylum claim	3.3% (4/121)	3.2% (7/222)		4.2% (4/96)	3% (6/200)		2.6% (9/343)	2.4% (3/125)	
Non-EU with no asylum claim	9.9% (12/121)	1.8% (4/222)		9.4% (9/96)	2% (4/200)		3.3% (11/343)	4.8% (6/125)	
Non-EU undocumented migrants	27.3% (33/121)	27% (60/222)		30.2% (29/96)	27.5% (55/200)		25.1% (86/343)	22.4% (28/125)	
Non-EU refugees	2.5% (3/121)	0.4% (1/222)		2.1% (2/96)	1% (2/200)		1.5% (5/343)	2.4% (3/125)	
Non-EU legal Migrants	16.6% (20/121)	17.1% (38/222)		14.5% (14/96)	16% (32/200)		16.6% (57/343)	18.4% (23/125)	

*P- value determined by chi2 test

### Barriers to Healthcare Access

Among patients with mental disorders, 62.4% (63/101) responded that high healthcare costs were a reason for not utilizing regular services, followed by troubles in administrative procedures in 29.7% (30/101), lack of awareness of available healthcare service in 32.7% (33/101), and language and communication problems in 16.8% (17/101). In addition, 13.8% (14/101) of the patients also reported fear of arrest or deportation when consulting health services.

## Discussion

Our research examines the relationship between socio-demographic characteristics, social determinants, and mental disorders in a heterogeneous group of patients with limited access to regular health services, who attended three humanitarian clinics in Germany in 2021.

The most prevalent mental disorders in our study population were severe stress and adjustment disorders/PTSD, followed by depression, alcohol-related disorders, anxiety, somatoform and sleep disorders. When examining socio-demographic characteristics, our findings showed a high proportion of mental disorders in patients originating from non-EU countries, and among refugees, undocumented migrants, persons with no health coverage and persons living in insecure housing.

A 2014 German health survey on twelve-month prevalence rates in the general population showed a high prevalence (37%) of mental disorders in younger adults (18–34 years old) [18]. This align with our study findings, since the age group 20–39 years old was significantly associated with increased odds of mental disorders.

Depression, anxiety, suicidal behaviour, and substance misuse have been reported as the predominant mental disorders in young people [19–21]. Altered mental health in the young adult population has been linked to decreased social activities, low social support, increased family pressure, exposure to abuse and discrimination, strong feelings of social isolation, lack of financial reserve and multi-morbidity [21–23]. Our results tend to confirm these findings, since a high proportion of young participants faced severe stress and adjustment disorders (19.2%), and reported feelings of depression (54.8%), a lack of interest in daily activities (55.1%) and a lack of psychological support when needed from a trusted friend or family member (44.3%) on most days of the week.

### Migration

According to the International Organization for Migration (IOM) migrants in high-income countries suffer high rates of mental disorders [24]. A migrant´s pre-migration peri-migration and post-migration exposures, including social, economic, environmental, political, and cultural context, are important factors determining their health status and health seeking behaviours [25]. According to a randomized controlled trial on mental health services provided to migrants following settlement in high-income countries, refugees and migrants represent a priority population with unique mental needs [24].

The majority (79.9%) of patients attending the humanitarian clinics for the first time in 2021 had a migrant background, with roughly half of them originating from EU countries other than Germany, and the other half from non-EU countries. Originating from a non-EU country was the only factor significantly associated with higher odds of a mental disorder in our analysis, although the significant association waned when adjusting for other socio-demographic factors. The “healthy migrant effect” is a well-known phenomenon and suggests that recent migrants tend to be healthier than the host country population due to a variety of pre-selection filters [7]. However, their health tends to worsen over time and eventually matches that of the general population in the host country [25].

In our study we screened only a subgroup of the migrant population in Germany, which can be characterized as urban, and which eventually sought healthcare at a humanitarian clinic., and therefore, we cannot infer on prevalence in the overall background migrant population. However, we could show that among individuals with perceived health needs, a large share showed signs of perceived altered mental health or were already diagnosed with a mental disorder. It can be assumed that in studies on migrants that are based on cross-sectional baseline screening, mental disorders can often be overlooked, as they tend to surface at a later stage after arrival in a host country and clinical manifestations often wax and wane over time.

Despite Germany receiving immigrants for many decades, the German integration policy is generally considered exclusionist [26]. The entitlement to health services as a feature of basic human rights has been criticized as not fully respected in Germany [27–29]. One example of legislation that may be considered non-inclusive is the Asylum Seekers Benefit Act, which only entitles asylum seekers to health services in emergency cases or for acute and painful conditions during the first 18 months of their stay, which has received criticism from the United Nations (UN) [30]. Furthermore, Article 87 of the Residence Act [Aufenthaltsgesetz requires every state institution in Germany to report migrants who cannot provide a valid residence permit, to the police or migration authorities. As a result, contact to official institutions for any purpose, including claiming basic human rights, such as, health care, can result in deportation and prevents undocumented migrants from seeking the services they require.

### Housing situation

The pathways linking a person’s living environment, housing and mental health are multidimensional and very complex [31]. The majority of the patients with mental disorders in our study fell into a classification of homelessness. Mental disorders both precede and are a consequence of homelessness and persons experiencing homelessness (PEH) suffer a substantial burden of mental health conditions [32, 33]. A systematic review by Ayano et al. reported a 6.5 times higher prevalence of depressive symptoms in PEH than in the general population [34].

Homelessness is associated with experiences of previous domestic violence, sexual assault, childhood traumatic experiences and social isolation, which likely contribute to the high prevalence of mental disorders in this population [35]. Furthermore, PEH often suffer high rates of physical co-morbidities, which may lead to deteriorating mental health [34].

A systematic review and meta-analysis of observational studies on the prevalence of mental disorders among PEH in Germany showed a high prevalence (77.5%) of mental disorders in PEH compared to the general population [36]. Despite a low rate (5,9%) of mental disorders in our overall study population, our data analysis revealed a high proportion (81.8%) of mental disorders in PEH. The heterogeneity of prevalence estimations can be attributed to the heterogeneous composition of our study population, as well as the methodological limitations of studies, such as limited sample sizes in some subgroups or the use of questionnaire items as approximation of mental disorder [35]. Irrespective of the variability of reported prevalence rates across different vulnerable subgroups with limited access to health services, it has been shown that mental disorders occur more frequently in PEH which we confirm [37].

### Health care insurance coverage

A lack of healthcare insurance coverage may negatively affect health [38, 39]. A 2015 report on health reforms in the United States shows that uninsured adults are far more likely to defer, renounce or not receive healthcare at all as compared to individuals covered by insurance [40]. This can lead to a lack of, or late diagnosis of preventable or chronic conditions, which can worsen over time and result in a high risk of morbidity and mortality. Our study demonstrates that a large proportion of participants with mental disorders had no statutory or private health insurance. These findings are in line with another existing study carried out in 2013 in the US, describing the role of insurance and healthcare access in individuals with mental disorders [41]. Despite the exclusionism that has characterized the German immigration policy over decades, regular immigrants, and their families – irrespective of their nationality – are entitled to membership in the statutory health insurance when they are employed or recipients of social welfare. However, many members of this subgroup do not exert their entitlements [17, 26].

### Barriers to health care services

The most commonly reported barrier to accessing regular healthcare services in our study population was costs of health services, also among persons with mental disorders. Affordability of health services has been reported as one of the major barriers to access in previous studies undertaken in Europe, South Africa, and the US [42–44]. However, health insurance coverage is only one dimension in the barrier complex with regard to healthcare [45, 46].

Barriers can be conceptually grouped into 5 categories, which reflect the affordability, approachability, availability, acceptability, and appropriateness [47–50]. Our data covers in principle the categories affordability (cost) and approachability (bureaucracy, language). Our findings demonstrate that the majority of patients with mental disorders were below the threshold poverty line (95.6%), reflected by “high healthcare costs” as the most frequently reported barrier. Furthermore, almost half of the patients (47.5%) required an interpreter which is provided by the services of the organization running the clinics, but which is mostly not well established in regular health services. Our results are consistent with a study on refugee mental healthcare utilization, which addresses the personal and structural barriers of refugees with psychotherapeutic needs in Germany in 2021. Lack of information, language difficulties, stigma, and fear of exclusion (among nine different barriers surveyed), were the most reported barriers in this study [51].

Mental health-related stigma is a multidimensional problem, which imposes a great burden on those affected [42]. Stigma plays an important role in influencing patients’ perceptions and, in some cases, leads to moral injury, by initiating or exacerbating mental disorders [52]. Behavior can be distorted by cultural perceptions of illness and its management, resulting in variation between self-perceived and observed morbidity [53, 54]. It can be assumed that access to mental health care is limited by perceptions of stigma and anticipated discrimination and may result in poor health-seeking behavior.

### Limitations

Several limitations must be taken into account when interpreting the results of this study. The setting of three humanitarian clinics and a mobile unit run by a single non-governmental organization in three major German cities is prone to selection bias, and external validity may be hampered, for example when extrapolation is intended towards people with limited access to regular health services in rural or non-metropolitan areas and to even more marginalized populations who do not seek healthcare services. As an example, psychotic disorders were under-represented in the studied patient population, possibly because individuals need to actively self-refer to the participating clinics in our study. As a result, persons suffering from more severe forms of mental disorders and marginalization could have been excluded from our patient population, resulting in a selection bias. In addition, in the course of data collection, we cannot exclude a certain degree of social desirability bias when marginalized individuals talk to healthcare workers, about their living conditions for example. This may also have been the cause to missing data, which lead to changing denominators in the analyses. Moreover, patients are not systematically screened for mental disorders, and not all clinics have a designated mental health professional making the diagnosis. The focus of consultations, especially at first visit, is also often on the patient’s somatic complaints, on trust building, and first stabilization. Mental disorders are therefore probably more prevalent than what is documented by the healthcare professionals at the clinics.

### Strengths

We were able to analyse a substantial dataset on a neglected and difficult to reach population in Germany, one that has limited access to healthcare services. We are convinced that even in the light of the stated limitations, our results are important for social and health science researchers as well as decision makers and agents of healthcare interventions, as we can provide baseline information on mental health needs in this particular population.

## Conclusions

Achieving the global target of Universal Health Coverage remains a challenge in high-income countries such as Germany. Our study shows that a large share of people with limited access to regular health services shows a substantial need for mental health services. As a chronic condition, managing mental disorders is even more difficult to establish outside of regular services, where humanitarian clinics are merely filling a gap in serving basic health needs. Policy makers in Germany should ensure the inclusion of marginalized populations in healthcare provision with a human rights approach to health in order to address the mental health needs of this vulnerable group.

## Data Availability

The data generated and analysed during this study are available from the corresponding author on reasonable request.

## Abbreviations

EEA: European Economic Area
EU: European Union
ICD-10: International classification of Diseases, Tenth Revision
IOM: International Organization for Migration
PEH: Persons Experiencing Homeless
PTSD: Post Traumatic Stress Disorder
UN: United Nations
WHO: World Health Organization
